# RNAi mediated down regulation of *myo*-inositol-3-phosphate synthase to generate low phytate rice

**DOI:** 10.1186/1939-8433-6-12

**Published:** 2013-05-15

**Authors:** Nusrat Ali, Soumitra Paul, Dipak Gayen, Sailendra Nath Sarkar, Swapan K Datta, Karabi Datta

**Affiliations:** Plant Molecular Biology and Biotechnology Laboratory, Department of Botany, University of Calcutta, 35, Ballygunge Circular road, Kolkata, 700019 WB India; Division of Crop Science, Indian Council of Agricultural Research (ICAR), Krishi Bhavan, Dr. Rajendra Prasad Road, New Delhi, 110001 India

**Keywords:** *Myo*-inositol, *Myo*-inositol-3-phosphate synthase, Phytic acid, RNAi silencing, Seed phosphorus, ABA sensitivity

## Abstract

**Background:**

Phytic acid (InsP_6_) is considered as the major source of phosphorus and inositol phosphates in cereal grains. Reduction of phytic acid level in cereal grains is desirable in view of its antinutrient properties to maximize mineral bioavailability and minimize the load of phosphorus waste management. We report here RNAi mediated seed-specific silencing of *myo*-inositol-3-phosphate synthase (*MIPS*) gene catalyzing the first step of phytic acid biosynthesis in rice. Moreover, we also studied the possible implications of *MIPS* silencing on *myo*-inositol and related metabolism, since, first step of phytic acid biosynthesis is also the rate limiting step of *myo*-inositol synthesis, catalyzed by MIPS.

**Results:**

The resulting transgenic rice plants (T_3_) showed a 4.59 fold down regulation in *MIPS* gene expression, which corresponds to a significant decrease in phytate levels and a simultaneous increment in the amount of inorganic phosphate in the seeds. A diminution in the *myo*-inositol content of transgenic plants was also observed due to disruption of the first step of phytic acid biosynthetic pathway, which further reduced the level of ascorbate and altered abscisic acid (ABA) sensitivity of the transgenic plants. In addition, our results shows that in the transgenic plants, the lower phytate levels has led to an increment of divalent cations, of which a 1.6 fold increase in the iron concentration in milled rice seeds was noteworthy. This increase could be attributed to reduced chelation of divalent metal (iron) cations, which may correlate to higher iron bioavailability in the endosperm of rice grains.

**Conclusion:**

The present study evidently suggests that seed-specific silencing of *MIPS* in transgenic rice plants can yield substantial reduction in levels of phytic acid along with an increase in inorganic phosphate content. However, it was also demonstrated that the low phytate seeds had an undesirable diminution in levels of *myo*-inositol and ascorbate, which probably led to sensitiveness of seeds to abscisic acid during germination. Therefore, it is suggested that though MIPS is the prime target for generation of low phytate transgenic plants, down-regulation of *MIPS* can have detrimental effect on *myo*-inositol synthesis and related pathways which are involved in key plant metabolism.

**Electronic supplementary material:**

The online version of this article (doi:10.1186/1939-8433-6-12) contains supplementary material, which is available to authorized users.

## Background

In cereal grains, phytic acid (InsP_6_) is considered as the major source of phosphorus and inositol phosphates. Phytic acid is an acidic compound which is highly reactive and readily binds to divalent mineral cations forming mixed salt complexes known as phytate (Lott et al. 1995). In rice, about 80% of phytate accumulates in the aleurone layer and embryo of mature seeds (Ogawa et al. 1977), which is degraded by phytase during germination (Laboure et al. 1993; Barrientos et al. 1994). Degradation of phytate mediated by the action of phytase, releases the bound mineral cations, phosphorus and *myo*-inositol which are required for proper growth and development of seedlings (Raboy 2002). Moreover, the monogastric animals, due to lack of this phytase enzyme, cannot digest phytate efficiently, rendering the bound phosphorus and mineral cations (Fe^2+^, Zn^2+^, Ca^2+^) unavailable for absorption (Bregitzer and Raboy 2006). Therefore, various attempts have been made to develop low phytate crops which would facilitate improvement in the bioavailability of phosphorus and micronutrients.

In plants, phytate biosynthesis is believed to be of ancient evolutionary origin and it has been suggested to proceed via sequential phosphorylation of inositol phosphates (Majumder et al. 1997,2003; Loewus and Murthy 2000). The enzyme 1D-*myo*-inositol-3-phosphate synthase (MIPS, EC 5.5.1.4) catalyzes the conversion of glucose-6-phosphate to *myo*-inositol-3-phosphate, which is the first step of *myo*-inositol biosynthesis and also directs phytic acid biosynthesis in seeds (Suzuki et al. 2007). MIPS being an important enzyme in the biosynthesis of phytate is often regarded as the prime target for reducing phytic acid level in cereals (Raboy 2009). The activity and expression of *MIPS* gene has been well characterized in different crops (Johnson 1994; Ishitani et al. 1996; RayChaudhuri et al. 1997; Hara et al. 2000; Hegeman et al. 2001; Shukla et al. 2004). Moreover, the accumulation pattern of *MIPS* transcript in developing rice seeds (*RINO1*) in relation to phytate globoids have also been well established, which suggests that *myo*-inositol-3-phosphate synthase plays a crucial role in InsP_6_ biosynthesis during the developmental stages of rice seeds (Yoshida et al. 1999). Recent reports, demonstrating successful manipulation of *MIPS* gene expression using transgenic strategy, suggests that reduction in *MIPS* transcript levels in developing rice seeds leads to a substantial decrease in phytic acid content of seeds (Feng and Yoshida 2004; Kuwano et al. 2009). However, seed *myo*-inositol contents of low phytate rice were not considered in these studies which might have a negative impact, as the first step of phytic acid biosynthesis is also the rate limiting step of *myo*-inositol synthesis, catalyzed by MIPS (Keller et al. 1998; Donahue et al. 2010) and its product *myo*-inositol-3-phosphate, is the only known precursor for the de novo synthesis of *myo*-inositol (Raboy 2002; Panzeri et al. 2011). Hence it is expected that efficient silencing of *MIPS* gene expression might affect the levels of *myo*-inositol in the low phytate rice. *Myo*-inositol being central to inositol metabolism is an important cellular metabolite required for normal plant growth and development (Stevenson et al. 2000; Valluru and Ende 2011). In addition to this, *myo*-inositol also contributes to plant protection against salinity by restoring the turgor pressure and protecting cellular structures from reactive oxygen species stress (Loewus and Murthy 2000; Majumder and Biswas 2006). Therefore, any changes in the levels of *myo*-inositol due to perturbation of MIPS may lead to alteration of the compounds synthesized later in the pathway, which can further disturb the signaling mechanism that regulates plants responses to different environmental stresses (Stevenson et al. 2000; Downes et al. 2005).

Another important aspect of phytate reduction is associated with an increase in the amount of iron in seeds. As mentioned earlier, phytate due to the presence of negatively charged phosphate side groups has a strong potential to chelate divalent cations like Fe^2+^ (Borg et al. 2009). Iron accumulated in the protein storage vacuoles along with phytate is not available for absorption, which corresponds to the lower bioavailability of iron in cereals (Brinch-Pedersen et al. 2007). Hence, it is understood that if there is reduction in levels of phytate, then larger amount of iron that is not chelated will be available in the endosperm, which translates itself into higher bioavailability of iron.

In the present study we report RNAi mediated silencing of *myo*-inositol −3- phosphate synthase (*MIPS*) gene, catalyzing the first step of phytic acid biosynthesis in *indica* rice cultivar. We developed low phytate rice by down regulating *MIPS* gene expression tissue specifically, through the use of seed specific promoter, *Oleosin 18* (*Ole18*). Analysis at the molecular and biochemical level suggested reduction in phytate levels, along with an increase of available phosphorus. Moreover, we also considered the effect of silencing *MIPS* on *myo*-inositol synthesis, which revealed that seed *myo*-inositol levels of transgenic plants were lower as compared to wild type, which correlates with the increased ABA sensitiveness during germination. In addition, we also estimated the increase in iron content of milled rice grains, which was enhanced due to reduction of phytate in seeds. Further the agronomic traits of the transgenic rice plants have been compared with non-transgenic controls.

## Results

### Development of transgenic rice plants

**Figure 1:**
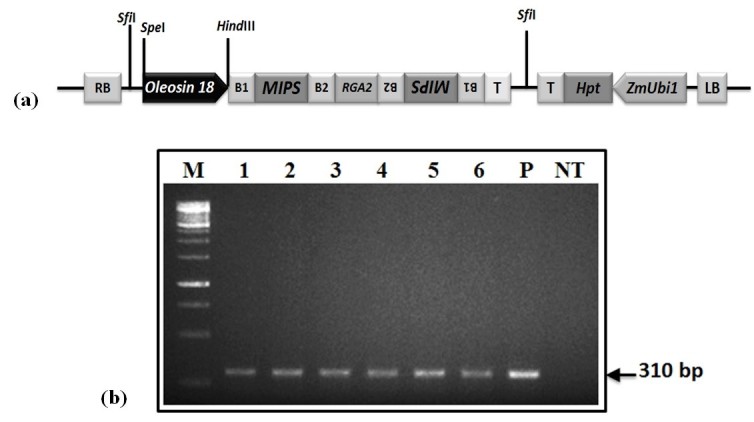
**Vector construct and molecular analysis of transgenic plants.** (**a**) Partial map of RNAi vector construct (p*Ole18*-*MIPS*-006) used for Biolistic transformation of *indica* rice cultivar, (**b**) gel picture of PCR analysis showing bands of *RGA2* intron as amplified from the transgenic rice plants. (M = 1 Kb gene ruler, P = Positive control, NT = Non-transgenic plant, lane 1-6 = Progenies of line MO6-196).

**Figure 2:**
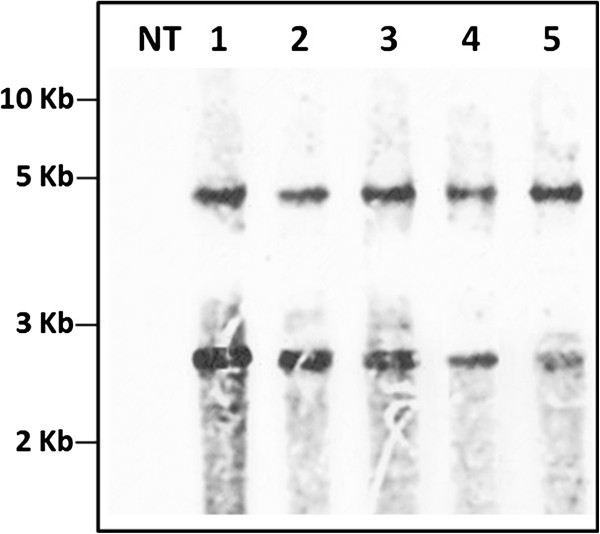
**Southern hybridization analysis of transgenic rice plants.** Genomic DNA (10 μg) of T_3_ progenies of line MO6-196 digested with *Eco* RI showing stable integration of *RGA2* intron in transgenic rice plants. The position and sizes of markers are indicated (NT = Non-transgenic plant, lane 1-5 = Progenies of line MO6-196).

In order to generate transgenic rice plants expressing a low phytate trait, we developed an RNAi vector construct into which fragment of rice *MIPS* gene was introduced following gateway based recombination system (Himmelbach et al. 2007). The vector (p*Ole18*-*MIPS*-006) (Figure 1a) was used for subsequent transformation of immature embryos, producing rice transgenic lines. The generated plants were screened by PCR analysis for the presence of transgene. PCR amplification of genomic DNA from 21 day old plants resulted in amplification of wheat *RGA2* intron only in transgenic positive plants while no amplification was observed in non-transgenic control plants (Figure 1b). Available Pi content of positive T_1_ seeds of individual transgenic lines was analyzed (Kuwano et al. 2009; Chen et al. 1956) and compared with the levels of non-transgenic rice seeds. The transgenic line MO6-196 exhibiting higher seed Pi levels was selected and subsequent generations (T_1_, T_2_, and T_3_) were grown similarly. Further, Southern hybridization analysis using T_3_ generation rice plants of line MO6-196 was performed, which revealed stable integration of the transgene cassette into the progenies (Figure 2). The Southern hybridization pattern was same for all the transgenic plants from the same examined line. The transgenic plants showed normal phenotype and were fertile.

### Morphological traits of transgenic plants

**Figure 3:**
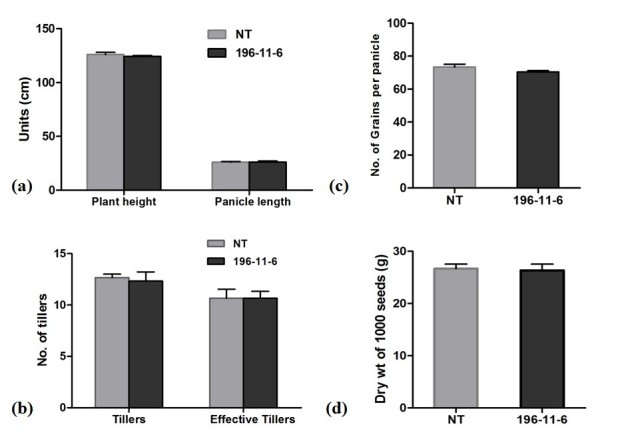
**Different agronomic characters analyzed in transgenic and non-transgenic rice plants.** (**a**) Plant height and panicle length (cm), (**b**) number of tillers and effective tillers, (**c**) number of grains per panicle and (**d**) 1000 seeds dry weight of non-transgenic and transgenic rice plants. No significant differences (P ≥ 0.05) were observed.

The morphological traits of the transgenic T_3_ plants were compared with non-transgenic control plants (Figure 3). In mature plants, there was no significant difference (P ≥ 0.05) in plant height and panicle length between T_3_ progenies of line 196-11-6 and non-transgenic control plants (Figure 3a). The number of effective tiller (Figure 3b), grains per panicle (Figure 3c) and 1000 seeds dry weight (Figure 3d) were also similar to non-transgenic control plants (P ≥ 0.05).

### Expression analysis of transgenic rice plants

**Figure 4:**
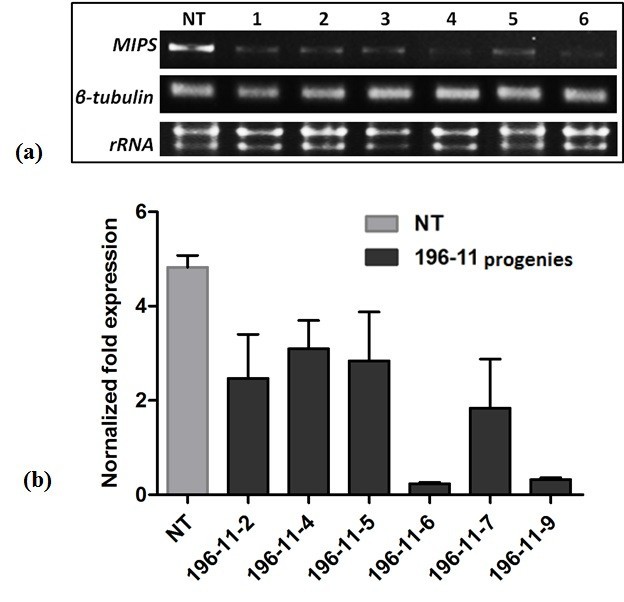
**Expression analysis of transgenic rice plants.** (**a**) RT-PCR analysis of T_3_ transgenic seeds of MO6-196, as compared to the internal control *β tubulin* reveals down-regulation in the transcript level of *MIPS* (**b**) Expression levels of *MIPS* as determined by Quantitative real time PCR. The normalized fold expression clearly indicates varied level of silencing, the maximum being 4.59 fold as observed in 196-11-6.

Rice seeds of both transgenic and non-transgenic control were analyzed by RT-PCR to detect the endogenous *MIPS* transcripts. Results revealed a distinct down-regulation in the expression of *MIPS* gene in transgenic plants with respect to non-transgenic control seeds (Figure 4a). However, all the seeds showed same level of expression for the house keeping gene, *β tubulin*. To further determine the down-regulation in expression of *MIPS* in transgenic rice seeds, with respect to non-transgenic control, quantitative real time RT-PCR (qRT-PCR) analysis was performed (Figure 4b). The expression profiles obtained, clearly indicates suppression of *MIPS* gene in transgenic rice. The normalized fold-reduction levels varied widely in the transgenic plants, the maximum reduction of 4.59 fold being observed in MO6-196-11-6.

### Seed phosphorus and phytic acid analysis

**Figure 5:**
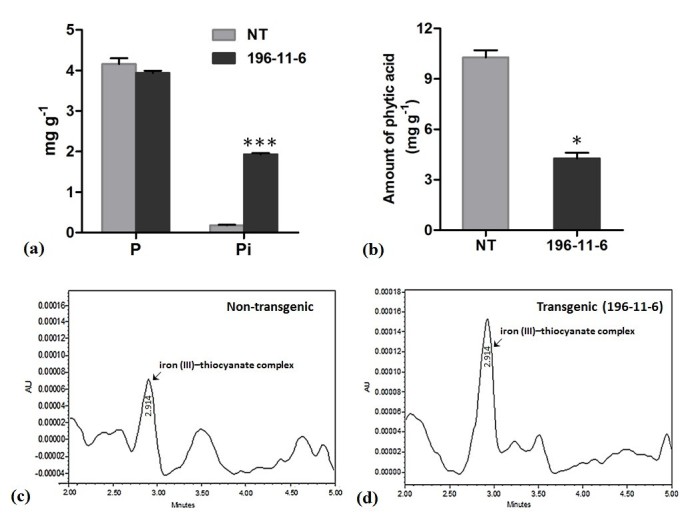
**Phosphorus and phytic acid content in seeds.** (**a**) Total phosphorus and Pi fractions in non-transgenic (NT) and T_3_ transgenic seeds and (**b**) amount of phytic acid in non-transgenic (NT) as compared to T_3_ low phytic acid transgenic seeds. The symbols * and *** indicates significant differences at P = 0.05 and 0.001 respectively. (**c** &**d**) HPLC traces showing peak of iron III- thiocyanate complex of non-transgenic and transgenic seed extracts of phytic acid after reaction with iron (III)- thiocyanate.

Total phosphorus levels in T_3_ seeds of transgenic and non-transgenic rice plants were determined. The average total phosphorus content of transgenic seeds was 3.939 mg g^-1^, which was not significantly different from that of non-transgenic seeds (4.162 mg g^-1^). Since, reduction in phytate level directly correlates with an increase of available phosphorus, Pi levels of transgenic seeds were analyzed, with respect to that of the non-transgenic seeds. The Pi concentration of transgenic rice seeds constitutes about 48.70% of the total phosphorus estimated in seeds, which was significantly higher than 4.33%, as observed in non-transgenic seeds (Figure 5a). Despite exhibiting higher Pi levels, the transgenic seeds displayed normal phenotype and did not show any aberration in embryo structure.

To further confirm reduction in phytate levels in transgenic seeds, phytic acid was quantified by HPLC (Waters, USA). The chromatogram obtained from HPLC/UV–vis method, at 460 nm showed larger peaks of iron (III)–thiocyanate complex, suggesting a significant decrease in the levels of phytic acid in transgenic seeds as compared to the non-transgenic control which exhibited smaller peak, signifying higher concentration of phytic acid in seeds. The amount of phytic acid, as calculated from the peak area was 10.28 mg g^-1^ for non-transgenic seeds and 4.273 mg g^-1^ for the seeds obtained from MO6-196-11-6, indicating a decrease in phytic acid levels by 58.43% in transgenic seeds (Figure 5b, c and d).

### Analysis of *myo*-inositol and metals in seeds

**Figure 6:**
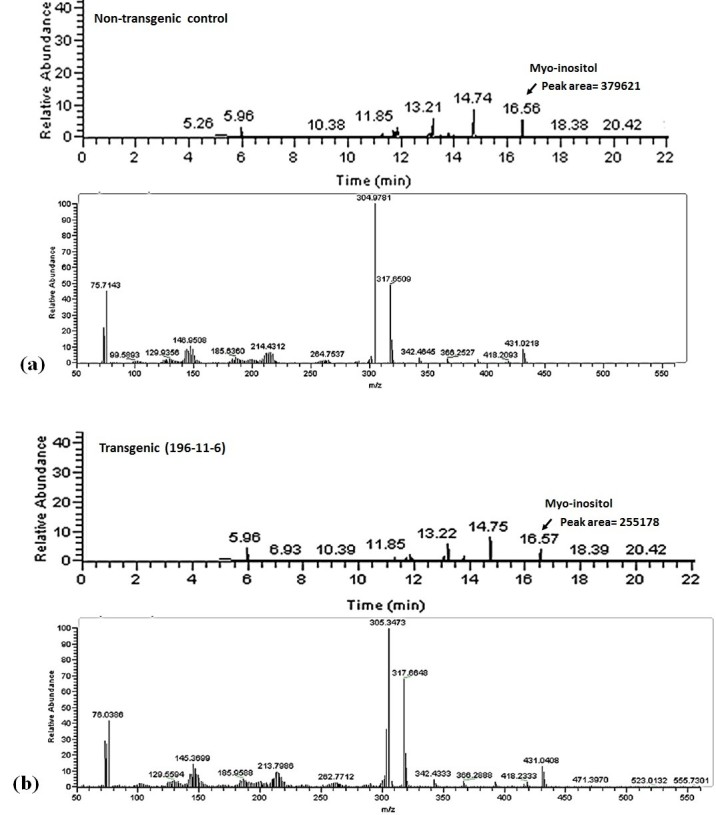
**Chromatogram showing peaks of** ***myo*** **-inositol (RT- 16.56) and respective mass fragments as observed by GC/MS analysis.** (**a**) Non-transgenic and (**b**) T_3_ transgenic seeds of 196-11-6. The peak corresponding to *myo*-inositol has been marked by an arrow. *Myo*-inositol was quantified as a hexa-trimethylsilyl ether derivative and was identified by comparing the mass fragmentation pattern with the database library NIST07.

The suppression of *MIPS* disrupts the synthesis of *myo*-inositol-3-phosphate, which is the precursor for de novo synthesis of *myo*-inositol. Hence it is expected that efficient silencing of *MIPS* gene expression would also reduce levels of *myo*-inositol in transgenic seeds. Therefore, we determined changes in *myo*-inositol content of transgenic seeds with respect to non-transgenic control, by GC/MS analysis. The data obtained from the analysis, strongly suggests that the amount of *myo*-inositol in transgenic seeds is reduced by approximately 28% as compared to the non-transgenic seeds (Figure 6).

Phytic acid chelates divalent metal cations mainly in the aleurone layer due to the presence of six highly negatively charged ions. So, in order to verify whether reduction in phytic acid levels have led to an increase in the amount of metal contents in the rice seeds, the content of different metal cations was measured in T_3_ transgenic and non-transgenic seeds (milled) by Atomic absorption spectroscopy (AAS, Perkin Elmer). The result clearly suggests an increase in the content of the divalent cations (Ca, Fe, Zn and Mg) measured (Table 1). Among the different metal cations analyzed, the amount of iron present in the milled rice grains of transgenic plants (11.62 μg g^-1^) was significantly higher than that of the non-transgenic milled seeds (7.027 μg g^-1^). The observations showed an increase of 1.3, 1.6 and 1.27 fold in the concentration of calcium, iron and magnesium respectively, due to the reduction of phytic acid in transgenic rice seeds (Table 1).

**Table 1:** **Metal content as analyzed by Atomic Absorption Spectroscopy from T**_**3**_ **milled seeds**

Metals	Non-transgenic	Transgenic
Calcium (μg g^-1^)	5.321 ± 0.067	7.196 ± 0.083
Iron (μg g^-1^)	7.027 ± 0.077	**11.62 ± 0.064**
Zinc (μg g^-1^)	22.30 ± 0.374	24.13 ± 0.135
Magnesium (mg g^-1^)	0.574 ± 0.010	0.732 ± 0.002

Values are mean ± SE, n = 3.

### Increased sensitivity to ABA during germination

**Figure 7:**
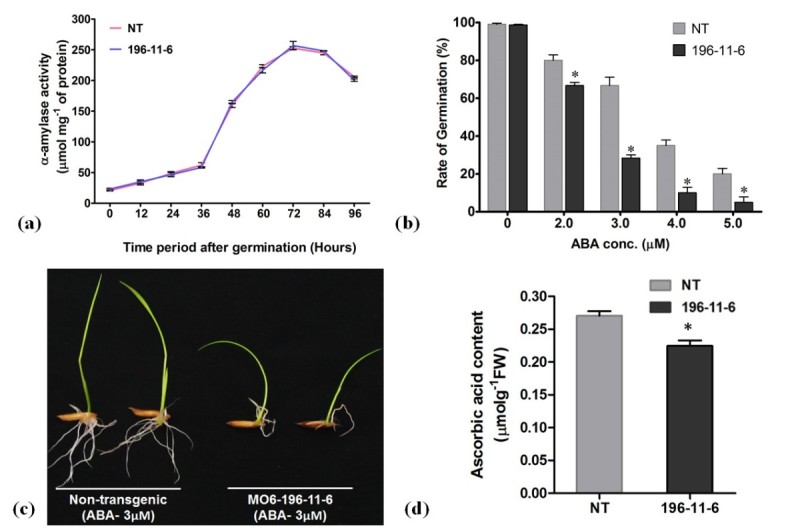
**Analysis during seed germination and estimation of ascorbate content.** (**a**) Alpha amylase activity analyzed at different time intervals after germination, (**b**) altered response of transgenic seeds during germination in presence of different concentrations of ABA, (**c**) picture showing increased sensitivity of transgenic seeds in presence of ABA (3 μM) at 7th day of germination and (**d**) Ascorbic acid content in non-transgenic (NT) and T_3_ transgenic rice seeds. The symbols * indicates significant differences at P = 0.05.

To verify the effect of reduced phytate levels on seed germination, which could have led to deleterious effects on plants, germination tests were conducted. No significant differences were observed in the rate of seed germination between the transgenic and non-transgenic control when grown in optimum conditions. Moreover, the α-amylase activities of transgenic seeds were also very similar to the non-transgenic ones (Figure 7a). As stated earlier, transgenic seeds exhibiting lower levels of *myo*-inositol might be correlated to their increased sensitivity to ABA during germination (Torabinejad et al. 2009; Donahue et al. 2010). Therefore, the transgenic as well as the non-transgenic seeds were germinated in presence of ABA. It was observed that in presence of ABA (3 μM), only 31.66% germination was recorded in transgenic seeds as compared to 63.34% of germination in non-transgenic control (Figure 7b and c). From the results it is clearly evident that though transgenic seeds display a normal germination pattern in favorable conditions, it is somewhat altered in response to ABA, which may correlate with the fact that decrease in *myo*-inositol content further reduces the ability of low phytate seeds to cope up with the reactive oxygen species (ROS) generated. The decrease in *myo*-inositol content also affected the ascorbate level in transgenic rice which was reduced by approximately 17% as compared to the non-transgenic control (Figure 7d).

## Discussion

In this study we demonstrated successful disruption of the first step of phytic acid biosynthetic pathway in transgenic rice plants, by silencing the gene expression of the enzyme *myo*-inositol-3-phosphate synthase. Earlier report suggests, higher suppression of *MIPS* gene expression could be achieved by using *Ole18* promoter, which directs expression specifically in the aleurone layer and embryo of rice seeds (Qu and Takaiwa 2004Kuwano et al. 2009). Hence, we generated transgenic rice plants, where *MIPS* gene expression was manipulated tissue specifically through the use of rice *Ole18* promoter, by an RNAi mediated approach. The transgenic plants produced, showed stable integration of the transgene cassette and displayed normal phenotype. In transgenic seeds the normalized fold expression of *MIPS*, at the transcriptional level suggests that silencing has been effective resulting in about 4.59 fold suppression of *MIPS* with respect to control. In view of previous studies, an obvious implication of silencing *MIPS* is the decrease in phytate levels (Raboy 2009), which was confirmed when the T_3_ generation seeds of line MO6-196-11-6 showed a 58% decline in the amount of phytic acid as compared to the non-transgenic control. In contrast to phytate, the Pi levels of transgenic seeds were enhanced by 44.37%, without disturbing the balance of total phosphorus in rice grains.

*Myo*-inositol-3-phosphate is an immediate precursor of *myo*-inositol, which is an essential metabolite known to play significant roles in signaling pathways, growth and plant development (Keller et al. 1998; Hegeman et al. 2001; Abid et al. 2009). Therefore, manipulating the gene expression of *MIPS* may result in subsidence in the level of *myo*-inositols. This was further confirmed by our results, which suggests a 28% decrease in *myo*-inositol of transgenic plants seeds. Recent reports revealed that *myo*-inositol biosynthesis is a highly regulated process which is involved in different biochemical pathways that are further associated with important metabolisms in plants (Seelan et al. 2009; Torabinejad et al. 2009). *Myo*-inositol has also been suggested to be the first metabolite of the alternative pathway synthesizing ascorbic acid, which is a powerful antioxidant and plays major role during rice seeds germination (Alimohammadi et al. 2012; Ye et al. 2012). Therefore, we analyzed the levels of ascorbate in transgenic plants which might have been affected due to lower levels of *myo*-inositol. It was observed that in transgenic plants, the ascorbic acid was decreased by 17% as compared to the non-transgenic control.

Low phytic acid phenotypes are often associated with downstream impacts on seed morphology and germination. From previous studies it is evident that reduction of phytate mediated by down regulation of *MIPS* might result in abortion of seeds (Nunes et al. 2006) or aberrant embryo structure (Kuwano et al. 2009) which may lead to impaired germination of the transgenic seeds. However, no such aberrations were observed in the transgenic seeds of subline MO6-196-11-6, inspite of having higher Pi levels. Earlier reports suggested that critical interference in the phytic acid biosynthesis might be lethal to seed germination due to reduction in phytate levels and simultaneous increase in the amount of Pi (Liu et al. 2007). But, the seed germination assay evidently suggests normal physiology of the transgenic seeds (MO6-196-11-6), which germinated in a similar pattern as that of non-transgenic control seeds, inspite of having elevated levels of available phosphorus. To further verify that the germination process was not impaired, we measured the activity of α-amylase (Bernfeld 1955), which is an important hydrolytic enzyme that catalyzes the breakdown of starch during germination and is also an indicator for assessing germination potential in cereals (Galani et al. 2011). The germination analysis of transgenic seeds displayed normal behavior of α-amylase suggesting that the seeds were viable, with normal phenotype. As revealed by prior reports, levels of *myo*-inositol in seeds due to its involvement in signal transduction correlates with their response to ABA during germination (Torabinejad et al. 2009; Donahue et al. 2010). Therefore, we observed the response of transgenic rice seeds to different concentrations of ABA during germination. The observations clearly indicates that reduced levels of *myo*-inositol, increases the sensitivity of transgenic seeds to ABA, as inhibition of germination was higher in low phytate seeds as compared to respective non-transgenic control seeds in presence of ABA. This increased sensitivity of transgenic seeds to ABA, might be attributed to decrease in the levels of ascorbate which is correlated to the probable decline of *myo*-inositol due to silencing of *MIPS* gene expression. Therefore, targeting other enzymes (IPKs) involved in phytic acid biosynthesis (Suzuki et al. 2007) may prove to be a promising alternative for producing lpa phenotype in rice seeds.

In rice plants, the metals such as iron, zinc, magnesium etc. are translocated to the reproductive organs via xylem and phloem through different types of transporters viz. ZIP, IRT, YSL etc. During seed development and maturation an elevated amount of Fe, Zn etc. are transported in the form of Nicotianamine acid or Mugineic Acid chelated complex (Paul et al. 2013; Lee et al. 2009). However, the accumulation of calcium in the developing seeds is nearly constant, as it is considered to move preferentially in xylem and is generally immobile in phloem which may be related to its symplastic nature (Iwai et al. 2012). Although sufficient amount of metals (Fe, Zn, Ca, Mg etc.) are translocated into the rice seeds, still in its milled form (that is consumed) rice contain very low amount of iron (Lucca et al. 2001). This is attributed to the fact that most of these divalent cations are chelated by phytic acid and accumulates in the aleurone layer and embryo (which is removed during commercial milling) as inclusions in protein storage vacuoles (globoids), which cannot move into the inner endosperm (Brinch-Pedersen et al. 2007; Raboy 2009; Iwai et al. 2012; Paul et al. 2013). Moreover, the iron molecules chelated by phytic acid is not bioavailable (Jin et al. 2009), probably due to lack of phytase enzyme in the non-ruminants (Hegeman et al. 2001). Therefore, it is assumed that lower level of phytic acid in the aleurone layer will allow more iron to be present in the endosperm which will thereby supply more available iron for absorption by humans.

In view of these assumptions we analyzed the amount of iron present in milled grains of low phytate T_3_ transgenic seeds, which showed a 1.6 fold increase in iron levels as compared to the non-transgenic rice. Apart from iron other divalent cations (viz. Ca, Mg) also showed higher accumulation in transgenic milled seeds, due to reduction of phytate. Although, the increase in levels of iron does not directly correspond to higher bioavailability of iron in humans, but in accordance with earlier studies (Lucca et al. 2001), it is assumed that elevated levels of iron in milled rice grains might translate itself to higher iron bioavailability. It is thus suggested that the reduction of phytic acid levels in transgenic rice grains may facilitate higher mobilization of iron towards inner endosperm in a chelated form with different types of Mugineic acid family derivatives such as NA (Nicotianaminic acid), DMA (Deoxy mugineic acid), etc. (Paul et al. 2013).

## Conclusion

Phytic acid constitutes 75-80% of the total phosphorus in cereal seeds but most of it is not accessible because the monogastric animals lack phytase, which is important for degradation of phytate molecule. This eventually leads to accelerate eutrophication due to the influx of phosphorus from animal waste which is the major source of agricultural phosphorus runoff (Reynolds and Davies 2001). Moreover, phytic acid readily binds to mineral cations and renders them unavailable for absorption by animals. Therefore, generation of low phytate rice is desirable for improving human nutrition and to reduce the load of phosphorus on environmental pollution. From the present investigation, it is evident that silencing of *MIPS* can yield major perturbation in the phytic acid biosynthetic pathway leading to substantial decrease in levels of phytate along with an increase in the amount of Pi content. However, the study also revealed that low phytate seeds had an unintended change in the levels of *myo*-inositol and ascorbate synthesis which is not desirable in view of its role in plants growth and development. Hence, it is clear that though *MIPS* presents itself as a candidate gene for manipulating phytate biosynthesis efficiently, silencing *myo*-inositol-3-phosphate synthase can yield major alterations in important metabolic pathways utilizing *myo*-inositol, which play key roles in different plant metabolisms. Therefore, a much detailed study of alterations caused in *myo*-inositol metabolism in view of disrupting *MIPS* expression, is required.

## Methods

### Plant materials and growth conditions

*Oryza sativa* L. subspecies *indica* cv. Swarna/IR-36 procured from Chinsurah Rice Research Station, Hooghly, West-Bengal, were used for cloning purposes. Rice seeds were surface sterilized and then washed 2–3 times with distilled water. The seeds were germinated on distilled water soaked filter paper within the plant growth chamber (FLI-2000, Eyela, Japan) maintained at 30°C and 75% relative humidity. For genetic transformation purpose *Oryza sativa* L. subspecies *indica* cv. Pusa Sugandhi II was available from IARI, ICAR, India.

### Cloning of rice *MIPS* gene and RNAi vector construction

Total RNA was extracted from *indica* rice cultivar using RNeasy Plant mini kit following manufacturer’s protocol (Qiagen). cDNA was synthesized from purified RNA using the Superscript III reverse transcriptase, two step RT-PCR kit (Invitrogen, USA) and gene specific primer pairs [see Additional file 1]. The RT-PCR product of *MIPS* gene [GenBank: AB012107] was purified and cloned into pENTR-D TOPO entry vector (Invitrogen) and sequenced. The 1.5 Kb fragment (111…1633 nt.) of *MIPS* gene from the entry clone (pENTR-*MIPS*) was then introduced into the binary destination vector, pIPKb006 (Himmelbach et al. 2007) using LR clonase (Invitrogen, USA) based recombination reaction. Finally, *Ole18* promoter [GenBank: AF019212] cloned from *indica* rice cultivar was subcloned into *Spe* I/*Hind* III site of pIPKb006 to generate the plasmid p*Ole18*-*MIPS*-006. The complete RNAi vector containing *MIPS* (in both sense and antisense orientation, separated by wheat *RGA2* intron) under the control of *Ole18* promoter (p*Ole18*-*MIPS*-006) was used for rice transformation experiments.

### Transformation and selection of transgenic plants

Biolistic transformation was carried out following the protocol described earlier by Datta et al. (1998). Immature embryos of *indica* rice cultivar Pusa Sugandhi II were bombarded with plant transformation vector constructs using Particle Delivery System (PDS-1000/He system, BIORAD, Hercules, CA, USA) following manufacturer’s instruction. Following bombardment, the immature embryos were transferred to the callus induction medium, supplemented with 50 mg l^-1^ hygromycin B (Sigma) and maintained in the dark at 27°C for 45 days. It was passed through three successive selection cycles of two weeks each. Transformed embryogenic calli resistant to hygromycin were selected and transferred to regeneration medium and maintained in 16/8 hour photoperiod at 28°C for 20 days. After development of proper root system, individual plants were transferred to the green house and grown to maturity.

### Southern hybridization analysis

Genomic DNA was isolated from positive T_3_ transgenic plants and non-transgenic control using DNeasy Plant mini kit following manufacturer’s protocol (Qiagen). Southern hybridization was performed, following the protocol as described by Sambrook and Russell (2001). Genomic DNA (10 μg) was digested with *EcoR* I (Fermentas), separated on a 1% agarose gel and transferred to a nylon membrane (Hybond N+, Amersham, GE Healthcare). Hybridization was carried out using the *RGA2* intron present in the vector p*Ole18*-*MIPS*-006, which was labeled with α-^32^P dCTP radioisotope (BARC, India), using Decalabel DNA labeling kit (Fermentas) according to the manufacturer’s instructions.

### Quantitative RT-PCR expression analysis

The qRT-PCR reaction was performed with gene specific primers (In*MIPS* F: 5′-CTTTCCGCACCTCAAACATT-3′; In*MIPS* R: 5′-TGCTGTCTCCAACATACGG-3′) using SYBR Green (Fermentas) and the cycle was as follows: 95°C for 30s, 59.6°C for 30s and 72°C for 30s. The procedure was according to the manufacturer’s instructions (CFX 96 Real time system, Bio Rad). The quantitative variation between different samples was evaluated by the ΔΔCt method, and the amplification of *β tubulin* gene was used as internal control to normalize all data. To validate the results, each experiment was performed in replicates on three separate RNA from independent tissue samples.

### Analysis of seed phosphorus levels

Total phosphorus in seeds was extracted by the alkaline peroxodisulphate digestion method (Woo and Maher 1995). The seed samples were crushed and to it 2 mL of digestion reagent (0.27 M potassium peroxodisulphate/0.24 M sodium hydroxide) and ten millilitres of deionized water were added. The sample mixture was autoclaved at 120°C for 60 min. One millilitre of the extract was then centrifuged at 20,000 *g* for 10 min, followed by spectrophotometric assay (Chen et al. 1956).

To analyze inorganic phosphate (Pi) levels, T_3_ transgenic seeds were ground to powder. The crushed powder was extracted in 12.5% (w/v) trichloroacetic acid containing 25 mM MgCl_2_, and centrifuged at 20,000 *g* for 10 min. Inorganic phosphate (Pi) in the supernatant was determined using 4 ml of freshly prepared Chen’s reagent (6 N H_2_SO_4_, 2.5% ammonium molybdate, 10% ascorbic acid). The resulting colored phosphomolybdate complex was read at 800 nm (Chen et al. 1956).

### Determination of phytic acid by HPLC

Chromatographic determination of phytic acid was based on metal replacement reaction of phytic acid from colored complex of iron (III)–thiocyanate and monitoring decrease in concentration of colored complex, if any (Dost and Tokul 2006). In a 3 ml glass tube, 0.1 ml of sample extract was mixed with 0.9 ml ultra-pure water and 2 ml of iron (III)–thiocyanate complex solution (100 ml iron(III)–thiocyanate solution was prepared by mixing 2.5 mg iron(III), 12.5 mg ammonium thiocyanate and 0.2 ml HNO_3_). The mixture was stirred in 40°C water bath for 2.5 h then cooled at room temperature. After centrifuging the mixture for 5 min, 20 μl of the supernatant was injected onto the column of the reverse phase HPLC system (Waters, USA). The mobile phase was a mixture of 30% acetonitrile in water including 0.1 M HNO_3_ and flow was adjusted to 1 ml min^−1^. The peak of the iron (III)–thiocyanate was detected at wavelength of 460 nm. Phytic acid concentration of the seed sample was calculated by using a linear calibration curve of y = −7.9667× + 1826.9 with correlation coefficient of 0.998 obtained against the concentration range of 10 μg ml^-1^ to 125 μg ml^-1^ of phytic acid standard (Sigma Aldrich; P0109) [see Additional file 2].

### Analysis of seed *myo*-inositol

T_3_ transgenic seeds were ground to powder, and extracted with 10 vol of 50% aqueous ethanol. The *myo*-inositol derivative was produced by dissolving the residues in 50 μl of pyridine and 50 μl of trimethylsilylimidazole: trimethylchlorosilane (100: 1). After 15 min at 60°C, 1 ml of 2,2,4-trimethylpentane and 0.5 ml of distilled water were added, the sample was vortexed and centrifuged for 5 min, and the upper organic layer was transferred into 2 ml glass vial (Panzeri et al. 2011). *Myo*-inositol [see Additional file 3] was quantified as a hexa-trimethylsilyl ether derivative by GC-MS (Trace GC Ultra, Thermo Scientific). Samples were injected in split mode (split ratio 10) with the injector temperature at 250°C and the oven at 70°C. After 2 min, the oven temperature was ramped at 25°C min^-1^ to 170°C, then continued to 215°C at 5°C min^-1^ and finally increased to 250°C at 25°C min^-1^ and returned to the initial temperature. Electron impact mass spectra from m⁄ z 50–500 were acquired at −70 eV after a 5 min solvent delay. *Myo*-inositol hexa-trimethylsilyl ether was identified by comparing the mass fragmentation pattern with the database library NIST07 (MS Library Software, Thermo Scientific). Authentic *myo*-inositol standards in aqueous solutions were dried, derivatized and analyzed at the same time. All analyses were performed in replicates.

### Metal concentration analysis

Mature T_3_ transgenic and non-transgenic seeds (grown in similar greenhouse conditions) were milled in rice miller (Satake, Japan) for 30 seconds. In this milling procedure, the outer portions of the rice grain including both germ and aleurone tissues were removed (degree of milling; D_OM_ was 6%). 300 mg of milled seeds were then digested using a modified protocol of dry ashing digestion (Jiang et al. 2007). The metal content (viz. Ca, Fe, Zn, Mg) of the sample extract was analyzed through Atomic Absorption Spectrometer (AAS, Aanalyst 200, Perkin Elmer, USA) using respective hollow cathode lamps (HCL, Perkin Elmer).

### Quantification of ascorbic acid

Ascorbic acid contents were measured in fresh imbibed rice seeds of both transgenic and non-transgenic lines, following the method described by Kampfenkel et al. (1995). The assay is based on the reduction of Fe^3+^ to Fe^2+^ with ascorbic acid in phosphoric acid solution followed by formation of red chelate between Fe^2+^ and 2, 2′-dipyridyl.

### Alpha amylase assay

Germinating seeds were collected at 0, 12, 24, 36, 48, 60, 72, 84 and 96 hours intervals and stored frozen at −80°C. In a pre-chilled mortar and pestle, the seed samples of both transgenic plants and non-transgenic control were crushed in 50 mM phosphate buffer (pH 7.0) and centrifuged at 4°C for 15 min. The enzyme assay was carried out with the supernatant, by incubating 100 μl of the enzyme extract with 1 ml of soluble starch (1%) at 50°C for 15 min. The reducing sugar released was estimated by addition of dinitrosalicylic acid (DNS) reagent (Miller 1959).

### Seed germination assay

The germination capability of T_3_ transgenic seeds as compared to non-transgenic control was assessed by controlled germination test (CGT) (Campion et al. 2009). In the CGT, seeds were soaked in water for 8 h at 30°C and then transferred to fresh water (CGT) at 30°C for additional 12 h. At the end of the treatment, seeds were rinsed two to three times in distilled water, and germinated on filter papers soaked with distilled water at 30°C in the dark. In addition to this, seed germination analysis was also performed in presence of different concentrations (0 - 5 μM) of ABA. The experiment was repeated thrice to confirm observations.

### Agronomic performance of transgenic plants

The different agronomic parameters like plant height (cm), panicle length (cm), number of effective tillers, number of panicles per plant and dry weight of 1000 grains were evaluated with both non-transgenic and transgenic plants. Five randomly chosen plants from each transgenic line growing under greenhouse conditions were evaluated for each parameter studied.

### Statistical analysis

All statistical analysis was performed using the Graph Pad Prism 5 software. The experimental data values were mean value from three independent series, each done with three replicates, and the results presented as means ± standard error (SE), based on three replications. Furthermore, the differences among means have been analyzed by Bonferroni Post-tests.

## Electronic supplementary material

Additional file 1: List of primers used for cloning of promoters and gene. (PDF 203 KB)

Additional file 2: Standard curve obtained from reference material (Phytic acid standard) for calculation of phytic acid concentration of seeds. (PDF 93 KB)

Additional file 3: *Myo*-inositol standard analysis by GC/MS for standardizing retention time. (PDF 150 KB)

Below are the links to the authors’ original submitted files for images.

Authors’ original file for figure 1

Authors’ original file for figure 2

Authors’ original file for figure 3

Authors’ original file for figure 4

Authors’ original file for figure 5

Authors’ original file for figure 6

Authors’ original file for figure 7

## Abbreviations

Pi: Inorganic phosphate
ABA: Abscisic acid
RNAi: RNA interference
ROS: Reactive oxygen species.
