# Supplementary material for: RNAi mediated down regulation of myo-inositol-3-phosphate synthase to generate low phytate rice
Source: Rice (N Y). 2013 May 15;6:12. doi: 10.1186/1939-8433-6-12 (PMC4883737; doi:10.1186/1939-8433-6-12)
Supplement: Supplementary file 1 — Additional file 1: List of primers used for cloning of promoters and gene. (PDF 203 KB) [file 12284_2012_48_MOESM1_ESM.pdf]

Additional file 1

Table 1: List of primers used for cloning of promoter and gene

Gene/Promoters	Forward	Reverse
Oleosin18	5’-TCAGCCAATACATTGATCCG-3’	5’-GCAAGATGAATGCAACGAAG-3’
MIPS	5’-TGTTCATCGAGAGCTTCCG-3’	5’-CTCCAGGATCATGTTGTTCTC-3’
RGA2 intron	5’-CCTGAAATTGGTAAAAGTAGA-3’	5’-TGTATCTTCATACTGCATTTG-3’
