# Supplementary material for: RNAi mediated down regulation of myo-inositol-3-phosphate synthase to generate low phytate rice
Source: Rice (N Y). 2013 May 15;6:12. doi: 10.1186/1939-8433-6-12 (PMC4883737; doi:10.1186/1939-8433-6-12)
Supplement: Supplementary file 2 — Additional file 2: Standard curve obtained from reference material (Phytic acid standard) for calculation of phytic acid concentration of seeds. (PDF 93 KB) [file 12284_2012_48_MOESM2_ESM.pdf]

Additional file 2

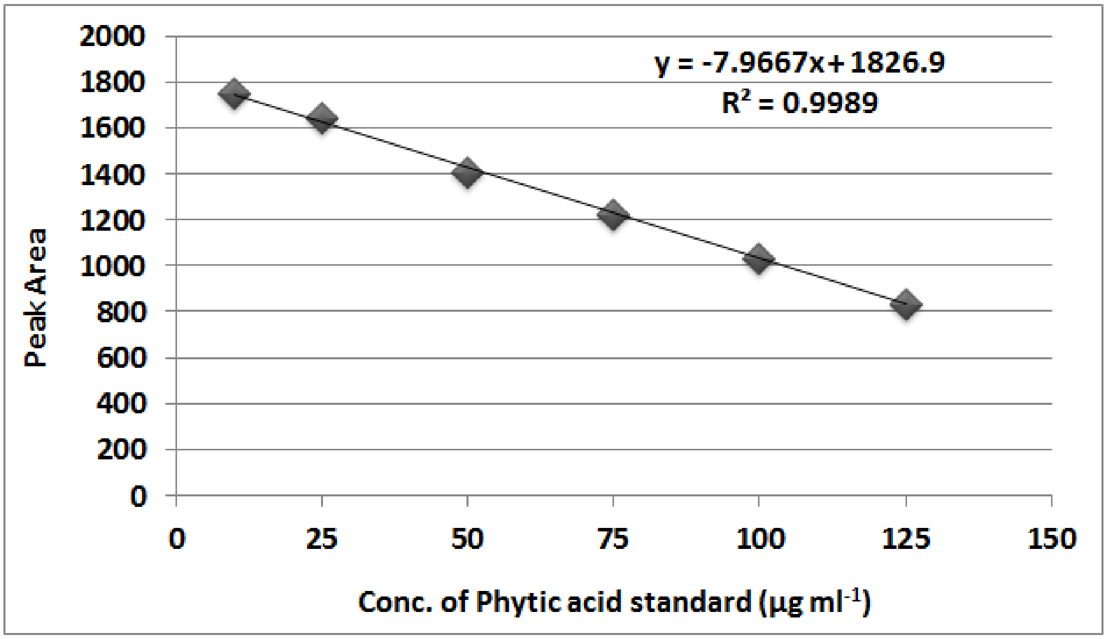

Figure: Standard curve obtained from reference material (Phytic acid standard) for calculation of phytic acid concentration of seeds.
