# Supplementary material for: RNAi mediated down regulation of myo-inositol-3-phosphate synthase to generate low phytate rice
Source: Rice (N Y). 2013 May 15;6:12. doi: 10.1186/1939-8433-6-12 (PMC4883737; doi:10.1186/1939-8433-6-12)
Supplement: Supplementary file 3 — Additional file 3: Myo-inositol standard analysis by GC/MS for standardizing retention time. (PDF 150 KB) [file 12284_2012_48_MOESM3_ESM.pdf]

Additional file 3

(A)

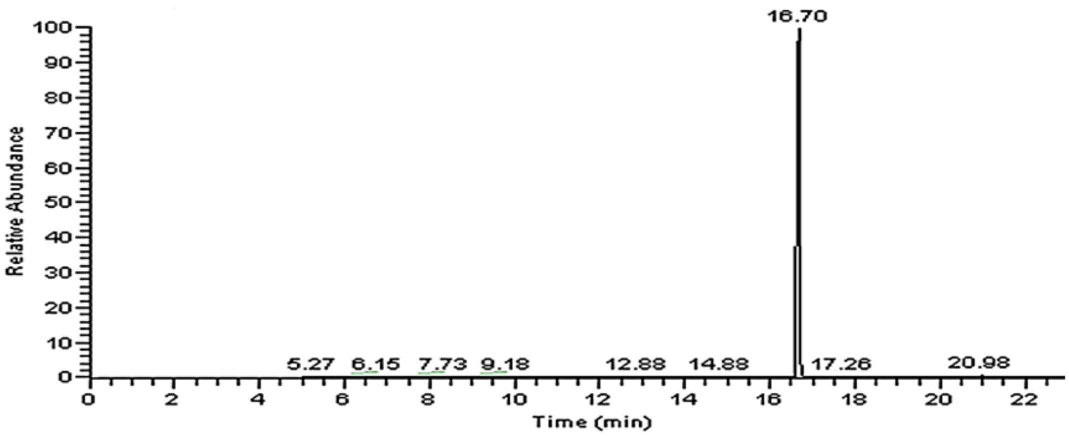

(B)

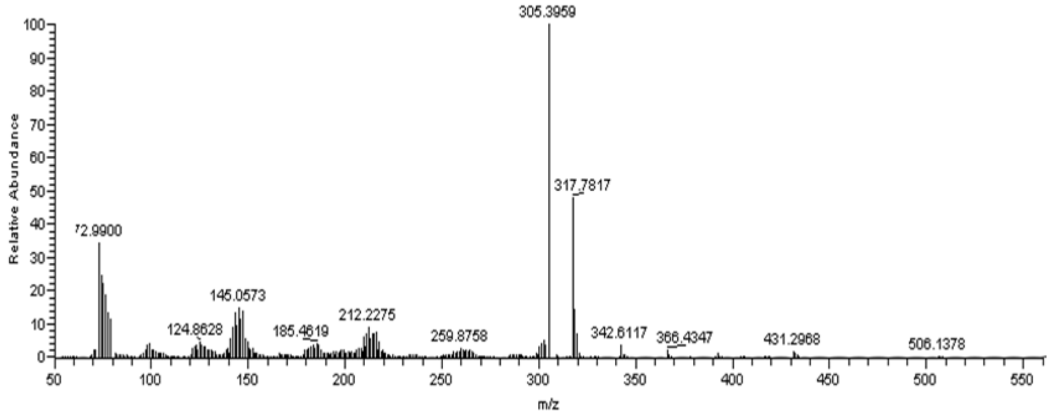

Figure: *Myo*-inositol standard analysis. (A) Chromatogram showing retention time of *myo*-inositol standard and (B) the respective mass fragmentation of *myo*-inositol peak.
