# Supplementary figures and images for: RNAi mediated down regulation of myo-inositol-3-phosphate synthase to generate low phytate rice
Source: Rice (N Y). 2013 May 15;6:12. doi: 10.1186/1939-8433-6-12 (PMC4883737; doi:10.1186/1939-8433-6-12)

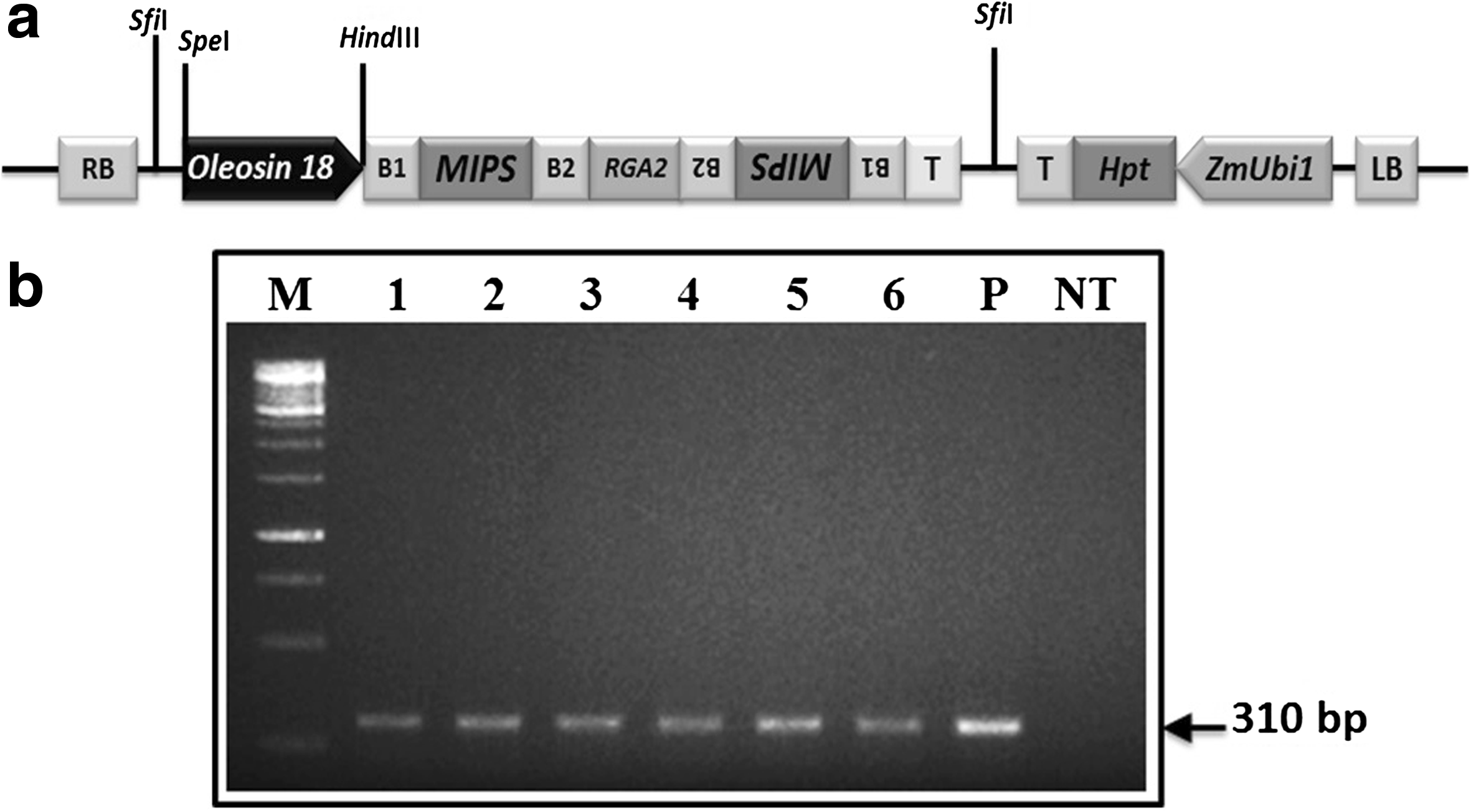

Supplement: Supplementary file 4 — Authors’ original file for figure 1 [file 12284_2012_48_MOESM4_ESM.tiff]

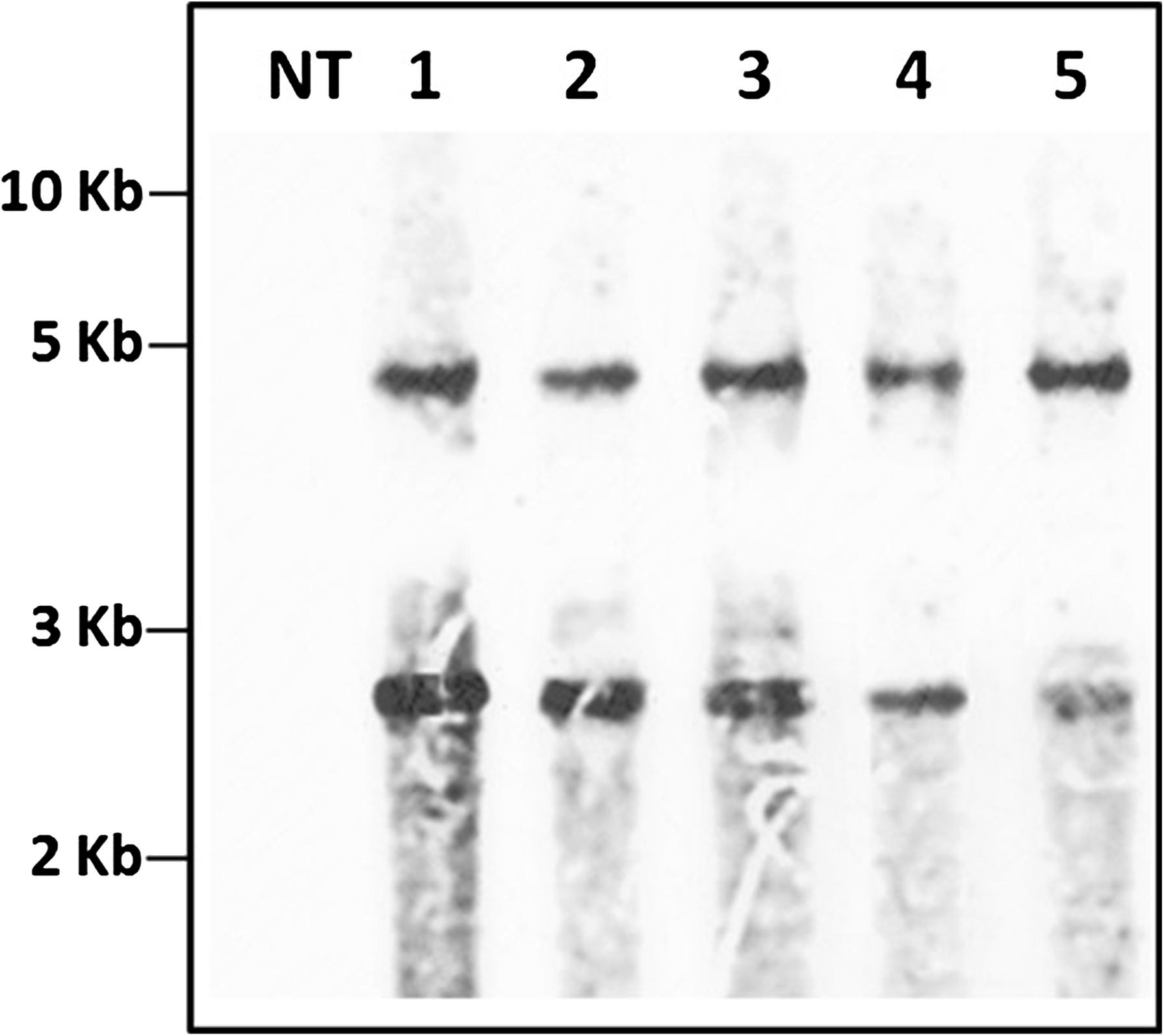

Supplement: Supplementary file 5 — Authors’ original file for figure 2 [file 12284_2012_48_MOESM5_ESM.tiff]

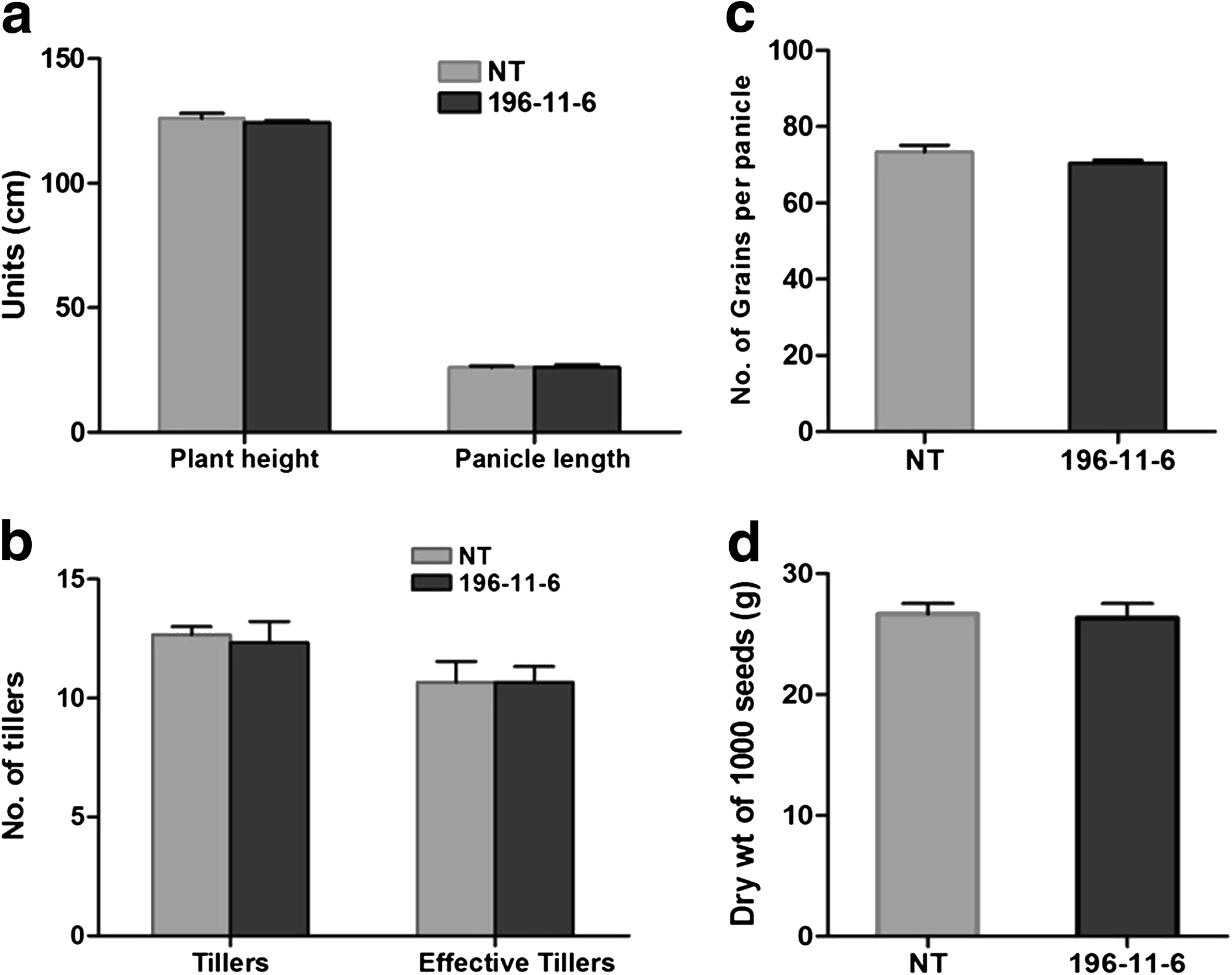

Supplement: Supplementary file 6 — Authors’ original file for figure 3 [file 12284_2012_48_MOESM6_ESM.tiff]

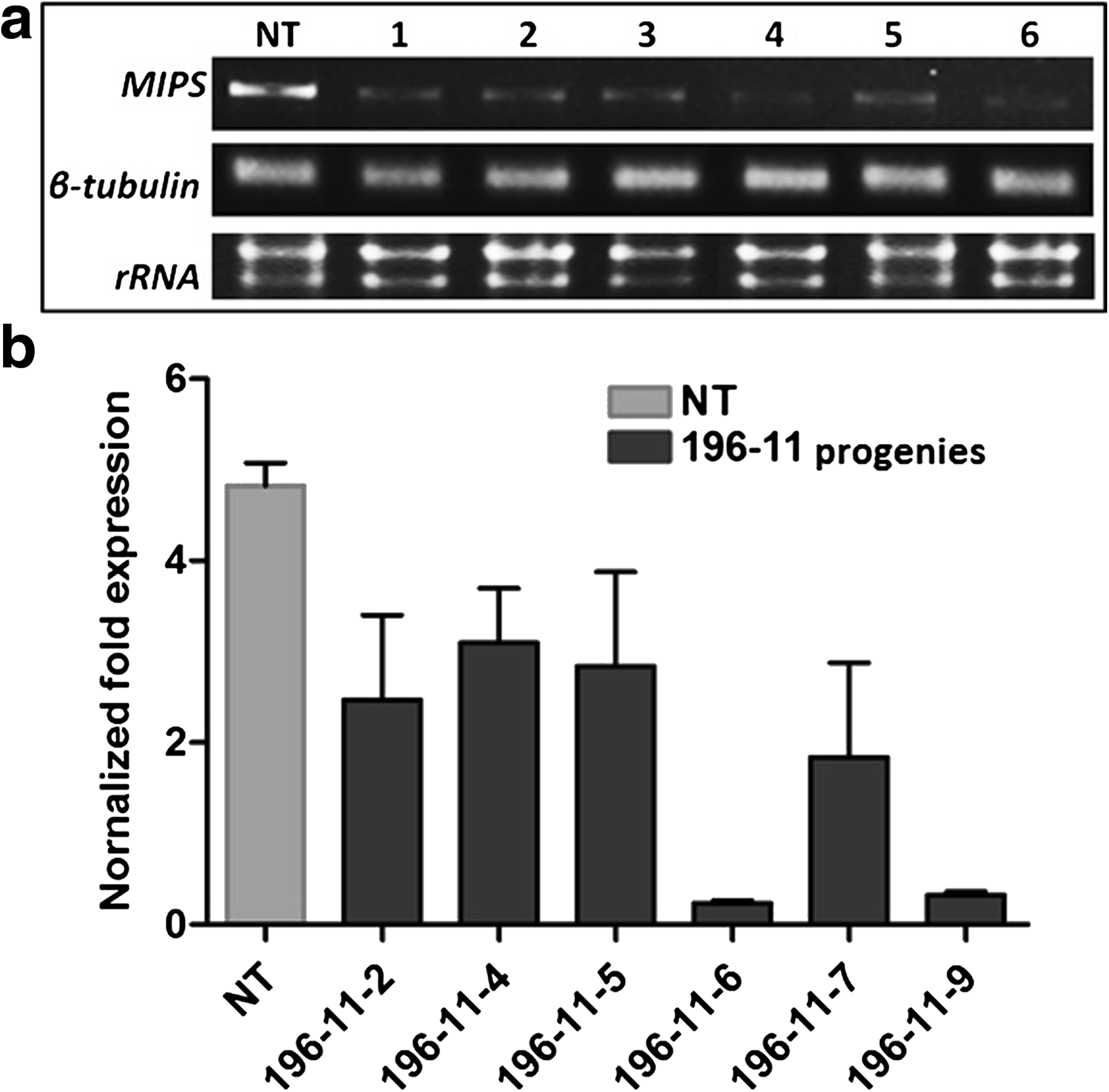

Supplement: Supplementary file 7 — Authors’ original file for figure 4 [file 12284_2012_48_MOESM7_ESM.tiff]

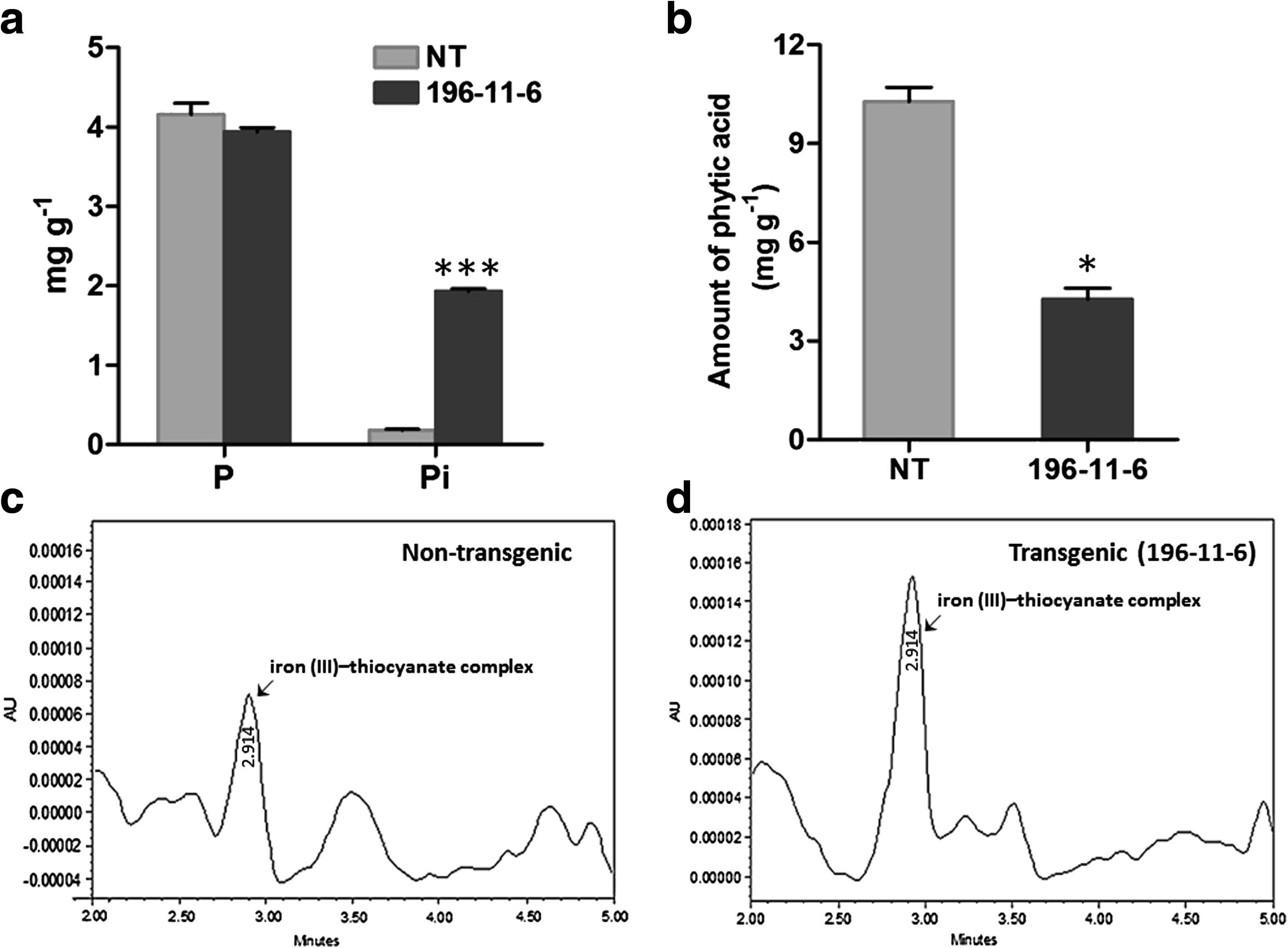

Supplement: Supplementary file 8 — Authors’ original file for figure 5 [file 12284_2012_48_MOESM8_ESM.tiff]

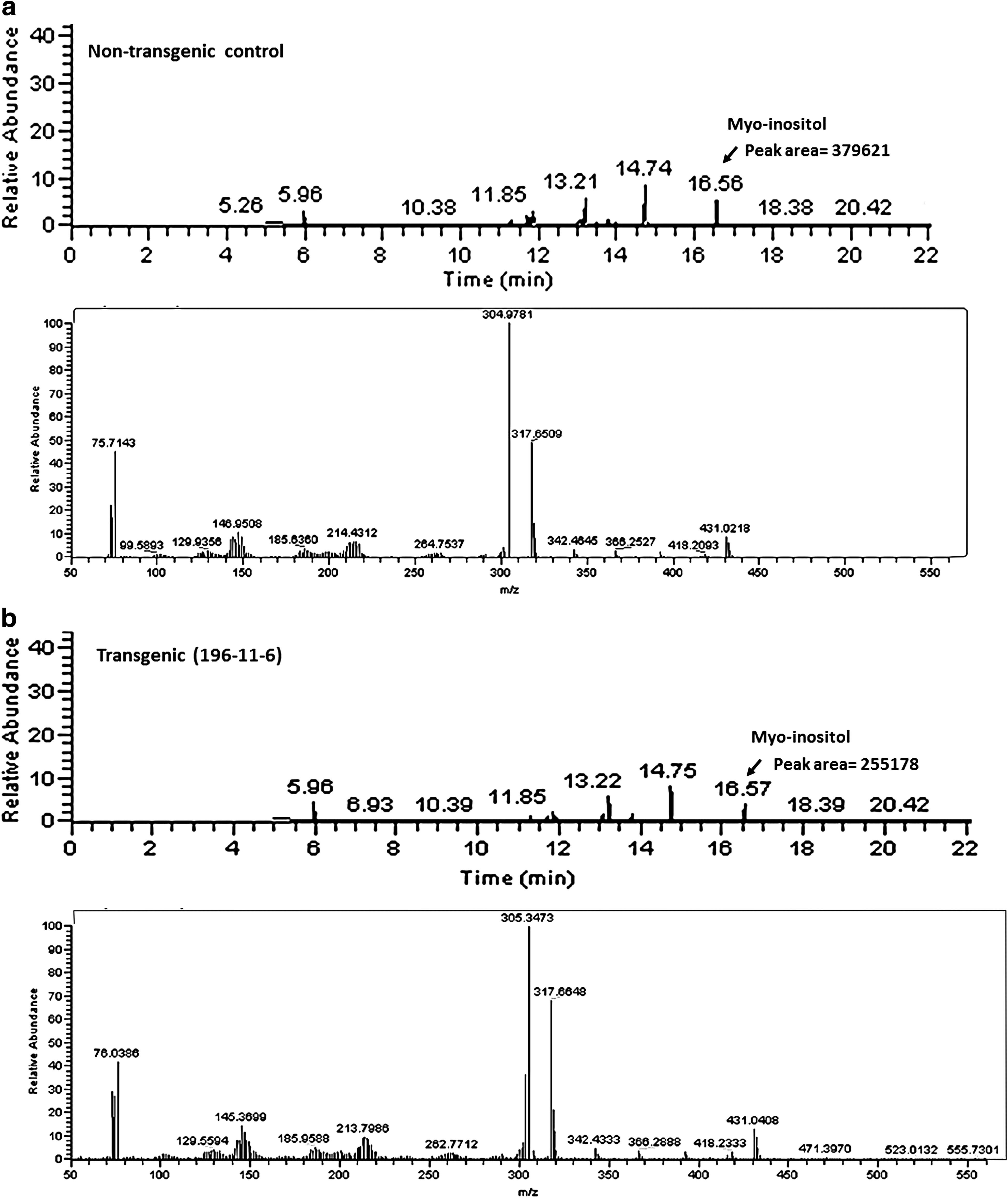

Supplement: Supplementary file 9 — Authors’ original file for figure 6 [file 12284_2012_48_MOESM9_ESM.tiff]

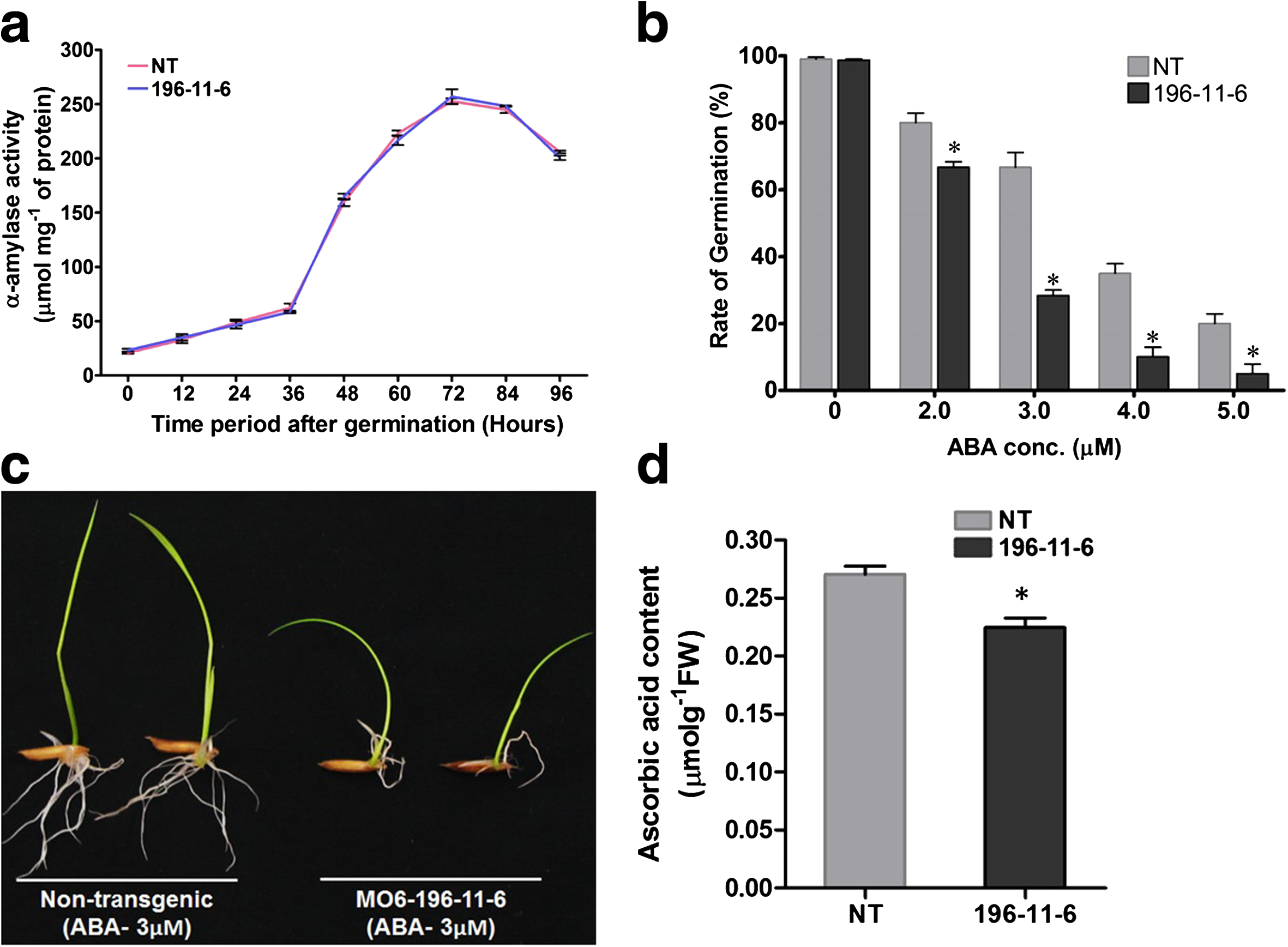

Supplement: Supplementary file 10 — Authors’ original file for figure 7 [file 12284_2012_48_MOESM10_ESM.tiff]
